# Early Epidemiologic and Immune Predictors of Atopic Dermatitis: Reduced Cord Blood Regulatory B10 Cells in the Munich Atopy Prediction Study (MAPS)

**DOI:** 10.1111/all.70306

**Published:** 2026-03-19

**Authors:** S. Preis, S. Kaesler, M. Koeberle, M. Hils, B. Evers, R. L. Silva, Y. Skabytska, L. Schellenberger, H. Hufnagel, M. Schielein, B. Kuschel, Y. Amar, A. Zink, T. Biedermann, Z. Kurgyis

**Affiliations:** ^1^ School of Medicine, Department of Dermatology and Allergy Technical University of Munich Munich Germany; ^2^ Institute for Medical Information Processing, Biometry and Epidemiology, Pettenkofer School of Public Health LMU Munich Munich Germany; ^3^ Department for BioMedical Research DBMR University of Bern Bern Switzerland; ^4^ School of Medicine, Department of Gynaecology, University Hospital Rechts der Isar Technical University of Munich Munich Germany

**Keywords:** atopic dermatitis, b cells, clinical immunology

## Abstract

**Background:**

A complex interaction between environmental and lifestyle factors, immune dysregulation, and skin barrier integrity is believed to contribute to the development of atopic dermatitis (AD). However, the precise mechanisms underlying disease onset in infants remain largely unclear.

**Methods:**

The “Munich Atopic Prediction Study” (MAPS) is a comprehensive clinical and biological investigation of a prospective birth cohort from Munich, Germany. Information on pregnancy, child development, environmental influences, parental exposure to potential allergens, as well as illnesses affecting both children and parents is gathered through questionnaires. This is complemented by thorough clinical examinations conducted by trained dermatologists, with a particular focus on allergies and skin health. Biomarker analyses were performed, for example, on cord blood immune cells using flow cytometry (FACS analysis).

**Results:**

Maternal AD (aOR = 3.06, *p* = 0.020) and affected siblings (aOR = 4.80, *p* = 0.039) are associated with an increased risk of AD, whereas cold‐remedy intake showed a protective association (aOR = 0.11, *p* = 0.047). Infants later diagnosed with AD (total 74 infants, AD *n* = 27, healthy *n* = 47) are characterized by reduced frequencies of CD4^+^ T cells (*p* = 0.0247) and increased B‐cell counts (*p* = 0.0067). Moreover, for the first time, we could identify a significant reduction in regulatory B (Breg)‐cell frequencies in these infants (*p* = 0.0015). Furthermore, our findings suggest that maternal allergen‐specific immunotherapy may have a beneficial effect on the development and frequency of Breg cells (*p* = 0.0497).

**Conclusion:**

Our study identifies early immune alterations, particularly a reduction in cord blood Breg cells, as potential contributors to AD pathogenesis. Incorporating Breg‐cell measurements into neonatal immune panels, along with key perinatal and familial risk factors, may enhance early risk stratification and enable more personalized prevention of atopic diseases.

## Graphical abstract

- In this prospective birth cohort from Munich, comprehensive clinical assessments and longitudinal lifestyle questionnaires were combined with cord blood immune profiling.
- Infants who later developed AD showed altered cord blood immune signatures, including reduced CD4+ T cells, increased B cells and markedly decreased Breg cells. Epidemiologic factors, such as maternal atopic dermatitis and siblings with AD were associated with increased while early cold‐remedy intake with decreased risk of developing AD.
- Maternal AIT was associated with increased neonatal Breg‐cell levels.

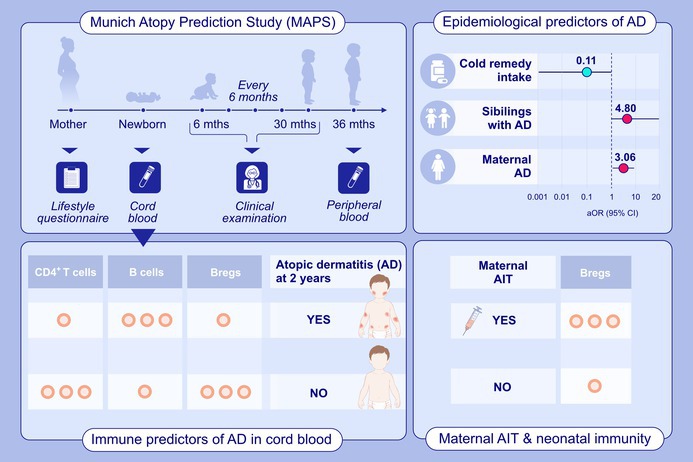

**Abbreviations**
AD: atopic dermatitis
AIT: allergen‐specific immunotherapy
aOR: adjusted odds ratio
Breg: regulatory B cell
CI: confidence interval
MAPS: Munich Atopy Prediction Study

## 1 Introduction

Atopic dermatitis (AD) is a chronic inflammatory skin disease that commonly begins in early childhood and affects 15%–20% of children and 2%–10% of adults in developed countries [1, 2]. It is characterized by poorly demarcated, pruritic, and scaling lesions and is associated with a considerable disease burden, impacting both quality of life and healthcare systems [3, 4, 5]. AD pathogenesis involves a multifactorial interplay of genetic predisposition, immune dysregulation, and environmental triggers, culminating in epidermal barrier dysfunction and chronic inflammation [6, 7].

Significant advances have been made in treatment strategies, including monoclonal antibodies targeting key cytokines such as interleukin‐4 (IL‐4), IL‐13, and IL‐31, as well as Janus kinase (JAK) inhibitors [8]. Early diagnosis and intervention are critical, as chronic inflammation and immune dysregulation in AD may predispose individuals to subsequent allergic conditions along the atopic march, including asthma and allergic rhinitis [9]. Identifying predictive biomarkers could enable risk stratification and early intervention, potentially through personalized application of emerging therapies [2, 10, 11]. Once clinical symptoms appear, AD can be diagnosed using validated criteria [10]. However, identifying individuals prior to symptom onset remains a challenge [10]. Prospective studies have begun to characterize the “preclinical” phase of AD, describing associated structural, immune, and microbial features [10].

A strong family history of atopic diseases is the most consistent risk factor [7, 12, 13]. The presence of one parent with AD increases the risk in offspring by approximately 37%, while two affected parents increase the risk to nearly 50% [14]. Genetic factors such as filaggrin (FLG) mutations play a pivotal role [15, 16, 17]. Filaggrin is essential for keratinocyte differentiation and barrier integrity [15, 16, 17]. Loss‐of‐function mutations, such as R501X, are associated with a 3.4‐fold increased risk of AD (OR 3.4; 95% CI: 1.7–6.8; *p* = 0.0002), with even higher risk in compound heterozygotes (OR 10.1; 95% CI: 4.7–22.1; *p* < 0.001) [18].

Environmental factors after birth also contribute significantly [7, 12, 13]. Urban exposures, including dust mite and cockroach allergens, indoor heating, cleaning products, and air pollutants—have been implicated in AD pathogenesis [19, 20]. Conversely, rural environments with greater microbial diversity may support the development of balanced immune responses that prevent allergic sensitization [21, 22]. Prenatal risk factors are more difficult to isolate but include maternal smoking, antibiotic exposure, gestational diabetes, and contact with industrial chemicals [19, 23]. During pregnancy, maternal immunity shifts toward a T‐helper 2 (Th2) profile, promoting fetal tolerance, a state that persists postnatally for several months [24, 25, 26]. Th2 cytokines, particularly IL‐4 and IL‐13, disrupt skin barrier function and are central to the inflammatory cascade in AD [27, 28, 29, 30, 31, 32]. While acute AD is characterized by Th2 dominance and elevated IL‐4, IL‐5, and IL‐13 levels, chronic AD may shift toward a Th1 response with interferon‐γ (IFN‐γ), exacerbating inflammation and barrier impairment [33, 34].

Much of our current understanding of AD pathogenesis derives from T‐cell‐focused research. In contrast, the role of B cells, particularly regulatory B cells (Bregs), has only recently gained attention [35, 36, 37]. Bregs are a heterogeneous population characterized by multiple subtypes, which are distinguished based on their surface markers, cytokine profiles, and stages of differentiation [37, 38]. The first identified Breg subset, the so‐called B10 cells, is defined by its IL‐10 production, although no unique surface markers exist [39]. B10 cells are enriched in CD19^+^CD24^hi^CD27^+^ and CD19^+^CD24^hi^CD38^+^ populations and are found in low frequencies in the spleen, peripheral blood, lymph nodes, intestinal tissues, and CNS [35, 40, 41, 42, 43]. B10 progenitors (B10pro) lack IL‐10 production at baseline but can differentiate into functional B10 cells upon stimulation, for example, via CD40 ligand over 48 h ex vivo [44, 45]. In this study, we use the term “Bregs” to refer to the phenotypically defined CD19^+^CD24^+^CD27^+^ regulatory B‐cell subset, whereas “B10 cells” specifically denotes IL‐10‐producing CD19^+^CD24^+^CD27^+^ B cells identified by intracellular cytokine staining. While known for their anti‐inflammatory IL‐10 secretion, B10 cells can, in principle, also secrete IL‐4, IL‐13, and TNF, cytokines linked to AD pathophysiology [46]. By suppressing effector T‐cell activity (especially Th2 and Th17), B10 cells promote immune regulation and may enhance expansion of regulatory T cells [39, 46, 47, 48]. Limited studies have explored B10‐cell involvement in AD [38, 49]. Gu et al. observed reduced B10‐cell frequencies in AD patients, though without a clear correlation to disease severity [38]. Yoshihara et al. confirmed reduced B10 levels in AD, with lower frequencies in patients with severe disease compared to those with milder forms [49].

Birth cohort studies offer valuable longitudinal insight by tracking immune development and environmental exposures from infancy [50, 51, 52]. Such studies have uncovered predictive markers for AD, including lower Th1:Th2 ratios and reduced regulatory T‐cell counts in cord blood [53, 54]. Moreover, maternal smoking during pregnancy is a risk factor for allergic diseases and has been correlated with multiple epigenetic changes and DNA methylation patterns in cord blood [55, 56]. To accurately interpret early‐life immune signatures, it is essential to place them within a well‐defined epidemiological context. Confirming established risk factors within our birth cohort ensures that the immunological findings are evaluated against a representative clinical background.

In this context, the Munich Atopic Prediction Study (MAPS) was established to identify novel predictive markers for AD. MAPS combines comprehensive biological sampling, including cord and peripheral blood, with questionnaire data from infants and their families. Unlike conventional cohorts, MAPS is designed for functional studies and longitudinal correlation with individual disease trajectories [11].

## 2 Methods

### 2.1 Subjects

MAPS is the comprehensive clinical and biological investigation of a prospective birth cohort from Munich, Germany focusing on a multifactorial characterization in the context of AD development in infants. A total of 375 infants were enrolled between May 2017 and March 2020 from three major hospitals. Inclusion criteria included maternal age ≥ 18 years, residence in the greater Munich area, and the ability to complete a German questionnaire. Cord blood was collected from 74 infants who completed follow‐up visits through age two. Of these, 27 infants developed AD, and 47 did not. AD diagnosis was based on clinical evaluation using Hanifin and Rajka criteria by a board‐certified dermatologist. Severity was assessed using SCORAD and EASI scores. The study protocol of MAPS has been described previously by Preis et al. [11]. The study was approved by the ethics committee of the Technical University of Munich and was in accordance with the Declaration of Helsinki (reference 334/16S).

### 2.2 Sample Collection and Clinical Data

Cord blood samples (80 mL) were collected immediately after birth from umbilical cord veins. Peripheral blood mononuclear cells (PBMCs) were isolated using Ficoll gradient centrifugation. Maternal sociodemographic and mental health data were collected prenatally and postnatally using standardized questionnaires. Clinical follow‐ups occurred at 2 months, 6 months, and 2 years. Skin examinations included detailed assessments of skin type, AD severity, and documentation of allergic symptoms, medications, and environmental exposures. Serum IgE concentrations were determined with the ImmunoCAP Total IgE assay on a Phadia 250 device (Thermo Fisher Scientific, Germany) according to the manufacturer's instructions.

### 2.3 Flow Cytometry and Immune Phenotyping

Multicolor flow cytometry panels were applied to identify immune cell subsets, including dendritic cells (CD11c, CD123, HLA‐DR, CD1c, CD14, Lin1), mast cell progenitors (CD34, CD133, CD13, CD117, Lin1), T cells (CD3, CD4, CD8, αβ‐TCR, γδ‐TCR), B cells (CD19), neutrophils (CD66b, CD33, CD123), myeloid‐derived suppressor cells (MDSCs) (CD11b, CD14, CD33, HLA‐DR, Lin1), and B10 cells (CD19, CD23, CD27, CD24, CD148, CD48). B10 cells were further characterized for their expression of IL‐10.

### 2.4 B10‐Cell Characterization and Functional Assays

PBMCs were cultured in RPMI 1640 medium with 10% FCS and stimulated with CpG ODN 2006, CD40L, PMA, and ionomycin. After 48 h, B10 cells were analyzed for IL‐10 expression via intracellular staining. Sorted CD19+ cells were recultured for IL‐10 secretion assays using ELISA. Additional markers assessed included PD‐L1, TIM‐1, CD1d, FAS‐L, PD‐1, TGF‐β, and Granzyme B.

### 2.5 Measurement of IL‐10 by ELISA and Multiplex Assay

IL‐10 concentrations were measured in serum using a commercial ELISA kit (Biolegend) following the manufacturer's instructions. In addition, IL‐10 was quantified using a bead‐based LegendPlex multiplex assay (LEGENDplex Human Th Cytokine Panel, BioLegend, Netherlands) and analyzed by flow cytometry.

### 2.6 Statistical Analysis

Univariate and multivariate logistic regression models were used to identify factors associated with the development of AD. Data were analyzed using SPSS, R, and GraphPad Prism. A *p*‐value < 0.05 was considered statistically significant.

Detailed protocols for the study itself, measurements of clinical data, PBMC processing, flow cytometry staining, B10‐cell culture and characterization, IL‐10 secretion assays, and statistical methods are provided in the Supplementary Methods.

## 3 Results

### 3.1 Clinical Characteristics of Infants Later Developing AD and Healthy Infants

Infants and mothers were enrolled in the Munich Atopy Prediction Study (MAPS), a birth cohort study in Munich, Germany (05/2017–03/2020) [11]. Of these, 345 infants were singletons and 15 were twin pairs (Figure S1). No significant sex difference was observed in AD prevalence (male 57.7%, *n* = 15 vs. female 42.3%, *n* = 11, *p* = 0.756). AD infants more often had affected mothers (38.5%, *n* = 10 vs. 17.2%, *n* = 31, *p* = 0.011) and siblings (15.4%, *n* = 4 vs. 3.3%, *n* = 6, *p* = 0.025) than healthy controls. No significant differences were found in residence (rural: AD 46.2%, *n* = 12 vs. healthy 28.3%, *n* = 51, *p* = 0.065) or season of birth (autumn‐winter: AD 73.1%, *n* = 19 vs. healthy 58.3%, *n* = 105, *p* = 0.051). Smoking prior to pregnancy was reported by 17.0% (*n* = 35) of mothers, with no significant group difference (AD 3.8%, *n* = 1 vs. healthy 18.9%, *n* = 34, *p* = 0.089). None of the AD infants received cold remedies in the first 6 months, while 12.2% (*n* = 22) of healthy infants did (*p* = 0.048) (Table S1).

### 3.2 Maternal AD and the Presence of Siblings With AD Increase the Risk of Developing AD, While Cold Remedy Intake Is Associated With Reduced Risk

Univariate analysis identified 5 of 24 markers associated with AD (*p* < 0.100, Table S2). Cold remedy intake was protective (OR = 0.12, 95% CI [0.001–0.89], *p* = 0.035), while maternal AD (OR = 3.02, *p* = 0.015) and sibling AD (OR = 5.37, *p* = 0.017) increased the risk for AD (Figure S2). Multivariate analysis confirmed maternal AD (aOR = 3.06, *p* = 0.020) and sibling AD (aOR = 4.80, *p* = 0.039) as major risk factors. Cold remedy use remained protective (aOR = 0.11, *p* = 0.047). Smoking had no significant effect (aOR = 0.24, *p* = 0.070) (Table 1). No multicollinearity was detected. The effect sizes of maternal AD, sibling AD, cold remedy intake, and maternal smoking prior to pregnancy showed general consistency between the models. No multicollinearity was observed between the predictors in the multivariate model (|Φ| ≥ 0.30, range: −0.24–0.19).

**TABLE 1:** Factors associated with AD at 6 months of life.

	Multiple logistic regression	
	aOR (95% CI)	*p*
Sex (male)	1.46 (0.62–3.60)	0.393
Maternal education (≥ 12 years)	0.67 (0.23–2.12)	0.478
Birth season (autumn‐winter)	1.74 (0.69–4.84)	0.247
Breastfeeding ≥ 6 months	1.30 (0.47–3.89)	0.611
Urban area	0.57 (0.24–1.42)	0.227
Cold remedy intake	0.11 (0.001–0.98)	0.047
Maternal AD	3.06 (1.20–7.68)	0.020
Siblings with AD	4.80 (1.09–20.81)	0.039
Mother smoking during the year before pregnancy (yes)	0.24 (0.02–1.11)	0.070

Abbreviations: AD: atopic dermatitis, aOR = adjusted Odds Ratio, aOR: adjusted odds ratio, bold values showed significance at alpha 0.05, CI: confidence interval, *n* = 206, MI: multiple imputation, OR: odds ratio.

### 3.3 Infants Developing AD Exhibit Lower CD4 ^+^ T‐Cell, Higher B‐Cell and Lower Breg‐Cell Frequencies in Cord Blood

**FIGURE 1:**
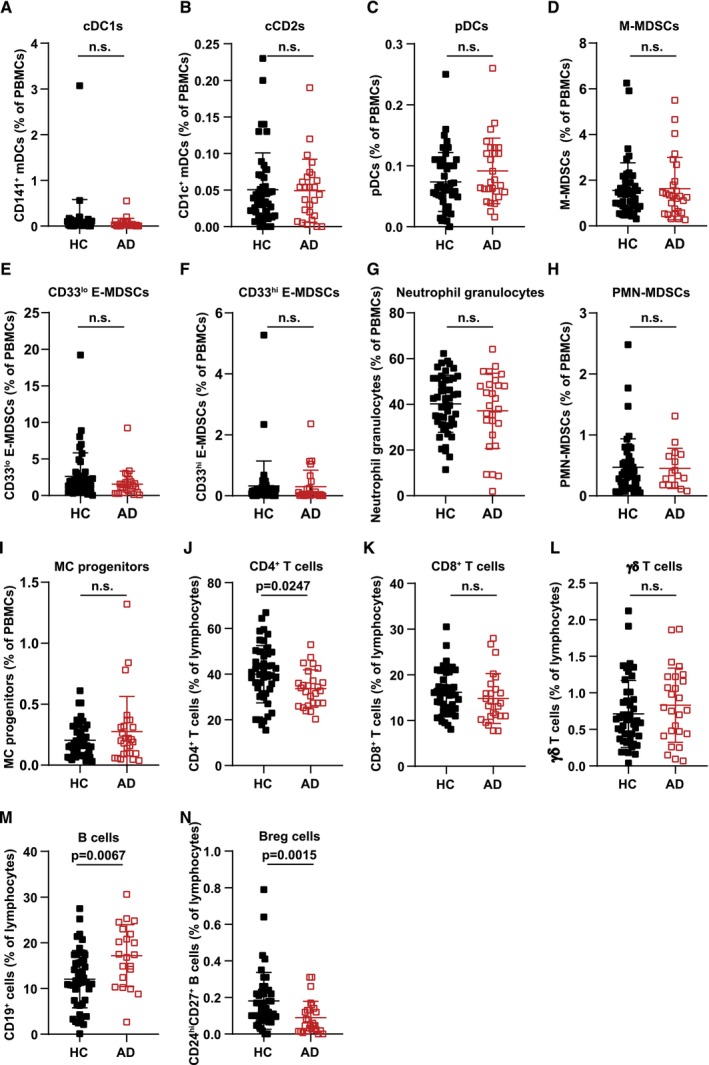
Infants developing AD showed lower cord blood CD4^+^ T‐cell, higher B‐cell and lower Breg‐cell frequencies. Frequency of (A) Lin1^−^HLA‐DR^+^CD14^−^CD11c^+^BDCA1^−^ cDC1 (B) Lin1^−^HLA‐DR^+^CD14^−^CD11c^+^BDCA1^+^ cDC2 (C) Lin1^−^HLA‐DR^+^CD14^−^CD11c^−^CD123^+^ pDC, (D) HLA‐DR^−^CD14^+^ M‐MDSC, (E) Lin1^−^HLA‐DR^−^CD11b^+^CD33^lo^ E‐MDSC, (F) Lin1^−^HLA‐DR^−^CD11b^+^CD33^hi^ E‐MDSC, (G) CD14^−^CD11b^+^ neutrophil granulocyte, (H) HLA‐DR^−^CD66b^+^CD33^+^CD123^+^ PMN‐MDSC, (I) Lin^−^CD34^+^CD133^+^CD13^+^ mast cell progenitors, (J) TCRα^+^TCRγ^−^CD4^+^CD8^−^ T cell, (K) TCRα^+^CD4^−^CD8^+^ T cell, (L) TCRα^−^TCRγ^+^ T cell, (M) CD19^+^ B‐cell and (N) CD19^+^CD24^hi^CD27^+^ regulatory B10‐cell populations in the indicated parent populations in the cord blood of infants later developing AD and in healthy infants. (A), C‐G, I: HC *n* = 47, AD *n* = 27; (B): HC *n* = 47, AD *n* = 26; H: HC *n* = 47, AD *n* = 16; (J–K): HC *n* = 46, AD *n* = 26, (M): HC *n* = 47 AD *n* = 21; (N): HC *n* = 46, AD *n* = 27. *p* values were calculated using Student's *t*‐test or Mann–Whitney test. Abbreviation: AD: Atopic dermatitis, HC: Healthy controls, NS: Not significant, cDC: Myeloid conventional dendritic cells pDCs: Plasmacytoid dendritic cells, M‐MDSCs: Monocytic myeloid‐derived suppressor cells, E‐MDSCs: Early‐stage myeloid‐derived suppressor cells, PMN‐MDSC: Polymorphonuclear myeloid‐derived suppressor cells; diagnostic certainty of AD based on 2‐year follow‐up.

To identify potential predictive biomarkers for the development or resistance to AD, we analyzed various immune cell populations in cord blood samples from infants with available 2‐year follow‐up data (Figure S3 and Figure 1). Of the 74 infants included, 27 developed AD within the first 2 years of life, while 47 remained unaffected.

No significant differences between the groups were observed in myeloid lineage immune cells, including conventional dendritic cells of the cDC1 (Figure 1A) and cDC2 subtypes (Figure 1B), total conventional dendritic cells, or plasmacytoid dendritic cells (Figure 1C). Similarly, analysis of immunomodulatory cell populations—including MDSCs, which we previously found increased in AD, and neutrophil subtypes, revealed no differences in M‐MDSCs (Figure 1D), CD33^lo^ early‐stage MDSCs (E‐MDSCs, Figure 1E), CD33^hi^ E‐MDSCs (Figure 1F), low‐density neutrophils (Figure 1G), or PMN‐MDSCs (Figure 1H). Mast cell progenitors were also present in comparable frequencies (Figure 1I) [57].

In contrast, analysis of lymphoid lineage cells revealed a significantly reduced frequency of CD4^+^ T cells in the cord blood of infants who later developed AD compared to healthy controls (*p* = 0.0247, Figure 1J), whereas CD8^+^ and γδ T‐cell frequencies did not differ significantly between groups (Figure 1K,L). When looking at diverse CD4+ T‐cell subsets, differences did not reach significance between infants who later developed AD and healthy controls (Figure S4). Notably, CD19^+^ B cells were significantly increased in infants developing AD (*p* = 0.0067, Figure 1M), while regulatory CD19^+^CD24^hi^CD27^+^ B cells were significantly reduced (*p* = 0.0015, Figure 1N), indicating an early imbalance between inflammatory and regulatory B‐cell subsets. Interestingly, total serum IgE concentrations in cord blood did not differ between newborns who later develop AD (*n* = 13) and HC (*n* = 28) (Figure S5).

### 3.4 Infants Developing AD Have Lower Breg‐Cell Frequencies in the Cord Blood Regardless of Maternal AD Status

**FIGURE 2:**
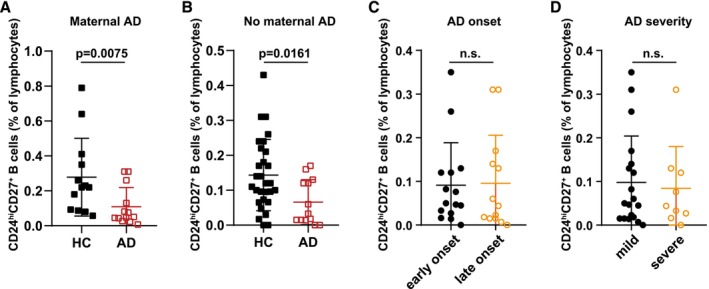
Frequencies of Breg cells were lower in the cord blood of infants developing AD independent of maternal AD. (A, B) CD19^+^CD24^hi^CD27^+^ Breg‐cell frequencies in infants developing AD and in healthy controls (A) with and (HC *n* = 13, AD *n* = 13) (B) without maternal AD (HC *n* = 29, AD *n* = 13). (C‐D) CD19^+^CD24^hi^CD27^+^ Breg‐cell frequencies in infants (C) with early (first 6 months of age, *n* = 15) and late (after 6 months of age *n* = 13) onset as well as (D) mild (EASI < 10, *n* = 19) and severe (EASI ≥ 10, *n* = 9) AD. AD: Atopic dermatitis, HC: Healthy controls, NS: Not significant. *p* values were calculated using Student's *t*‐test, diagnostic certainty of AD based on 2‐year follow‐up.

Cord blood predominantly contains fetal cells, though maternal cells can also be present [58]. Maternal immune status during pregnancy may influence the neonatal immune repertoire [59, 60]. Given that maternal AD is more common in children who later develop AD, we aimed to investigate whether maternal AD affects cord blood Breg‐cell frequencies. To assess this, we evaluated the percentage of CD19^+^CD24^hi^CD27^+^ cells in cord blood mononuclear cells (CBMCs) of infants born to mothers with and without AD. In both groups, we found a significantly lower percentage of CD19^+^CD24^hi^CD27^+^ cells in infants who later developed AD (*p* = 0.0075 in mothers with AD and *p* = 0.0161 in healthy mothers, Figure 2A,B). This suggests that the reduced frequency of regulatory B cells in cord blood is unlikely to result from maternal AD.

We next investigated whether reduced Breg frequencies were associated with earlier AD onset or disease severity. The observation period was 24 months. Comparing early (< 6 months) and later onset (> 6 months), no difference in CD19^+^CD24^hi^CD27^+^ cell percentages was observed (Figure 2C). Similarly, there was no significant difference between infants who developed mild vs. severe AD (defined as EASI < 10 or ≥ 10; Figure 2D).

### 3.5 Maternal Allergen‐Specific Immunotherapy Positively Correlates With the Frequency of CD19 ^+^ CD24^hi^CD27 ^+^ Breg Cells in Cord Blood

**FIGURE 3:**
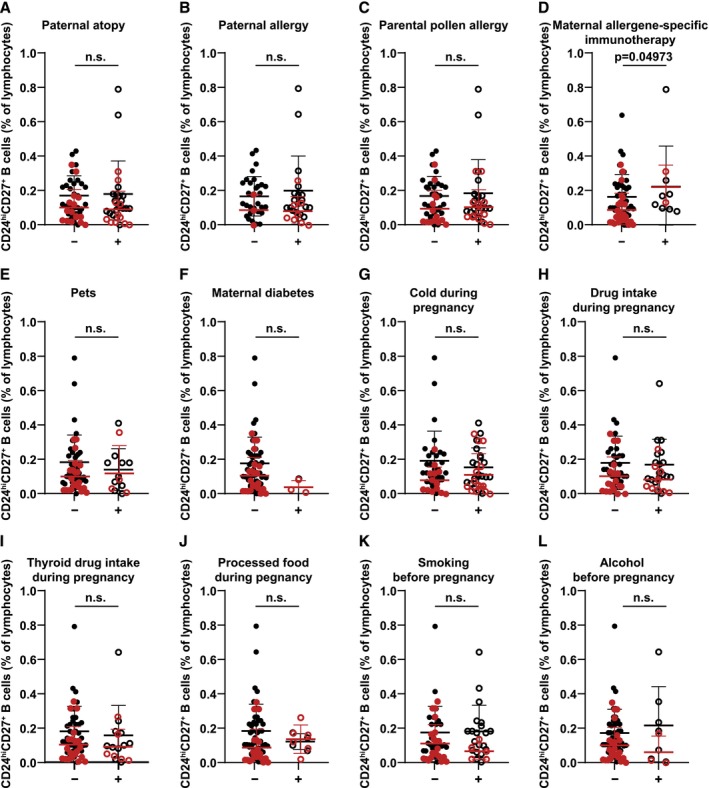
Positive correlation of maternal allergen‐specific immunotherapy with the frequencies of cord‐blood CD19^+^CD24^hi^CD27^+^ Breg cells. (A–L) CD19^+^CD24^hi^CD27^+^ Breg‐cell frequencies in the presence and absence of the indicated external factors *p* values were calculated using Student's *t*‐test, diagnostic certainty of AD based on 2‐year follow‐up. A: HC‐ *n* = 28, HC+ *n* = 21, AD‐ *n* = 16, AD+ *n* = 11; B: HC‐ *n* = 3, HC+ *n* = 18, AD‐ *n* = 3 AD+ *n* = 8; C: HC‐ *n* = 29, HC+ *n* = 20, AD‐ *n* = 14, AD+ *n* = 13; D: HC‐ *n* = 41, HC+ *n* = 8, AD‐ *n* = 25, AD+ *n* = 2; E: HC‐ *n* = 39, HC+ *n* = 10, AD‐ *n* = 23, AD+ *n* = 4; F: HC‐ *n* = 48, HC+ *n* = 1, AD‐ *n* = 24, AD+ *n* = 3; G: HC‐ *n* = 29, HC+ *n* = 20, AD‐ *n* = 13, AD+ *n* = 14; H: HC‐ *n* = 31, HC+ *n* = 18, AD‐ *n* = 18, AD+ *n* = 9; I: HC‐ *n* = 38, HC+ *n* = 11, AD‐ *n* = 21, AD+ *n* = 6; J: HC‐ *n* = 45, HC+ *n* = 4, AD‐ *n* = 2, AD+ *n* = 6; K: HC‐ *n* = 29, HC+ *n* = 20, AD‐ *n* = 22, AD+ *n* = 5; L: HC‐ *n* = 42, HC+ *n* = 7, AD‐ *n* = 24, AD+ *n* = 3. Abbreviation: Red: AD patients, black: Healthy controls.

To investigate what influences the frequency of Breg cells in cord blood, we analyzed various clinical, environmental, and paternal parameters collected in our study (Figure 3). No effect was observed for paternal factors such as paternal atopy, allergy, or pollen allergy on CD19^+^CD24^hi^CD27^+^ cell frequencies in cord blood (Figure 3A–C). Importantly, however, infants born to mothers who had received allergen‐specific immunotherapy (AIT, documented AIT: 7 SCIT and 3 SLIT, performed for pollen allergy (*n* = 8) and house dust mite allergy (*n* = 2)) prior to pregnancy showed slightly but significantly higher numbers of CD19^+^CD24^hi^CD27^+^ cells in cord blood compared to those whose mothers had not received AIT (*p* = 0.0497, Figure 3D).

Other factors, including pet exposure at home, maternal diabetes, common cold episodes, general or thyroid‐specific medication during pregnancy, dietary habits (home‐cooked vs. processed food), as well as smoking and alcohol consumption before pregnancy, did not significantly influence CD19^+^CD24^hi^CD27^+^ cell frequencies (Figure 3E–L).

### 3.6 CD19 ^+^ CD24^hi^CD27 ^+^ Cells Secrete IL‐10

**FIGURE 4:**
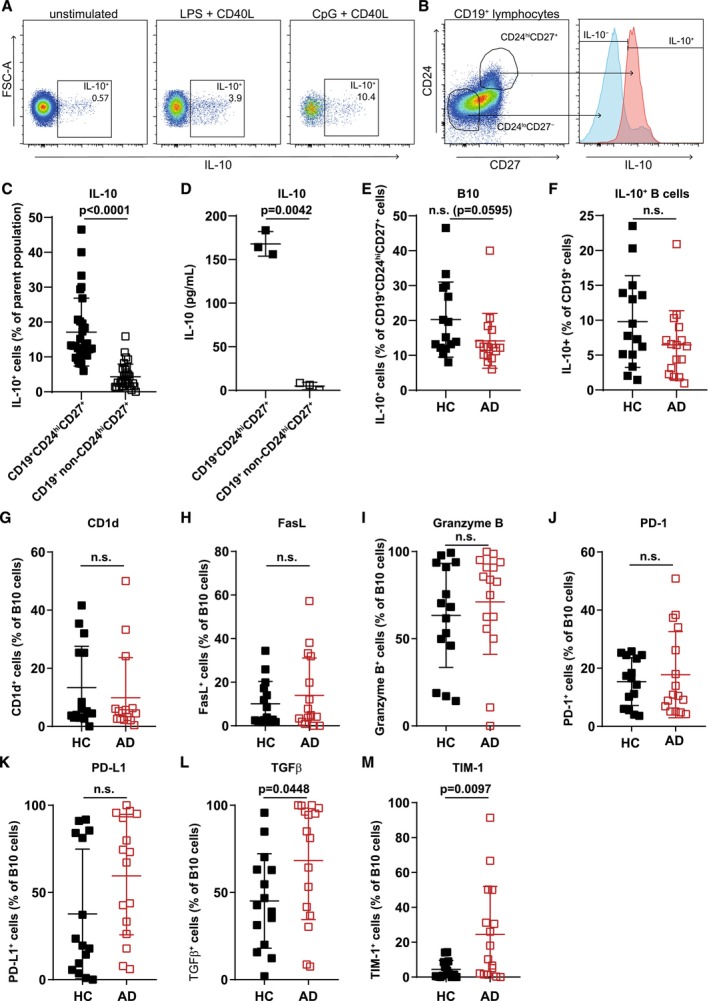
Cord blood derived B10 cells in infants later developing AD upregulate regulatory markers (A) Cord blood derived mononuclear cells were stimulated with LPS and CD40L or CpG and CD40L for 48 h. Frequencies of CD19^+^IL‐10^+^ cells measured by flow cytometry are shown. (B–C) Frequency of IL‐10^+^ cells among CD19^+^CD24^hi^CD27^+^ as well as CD19^+^CD24^lo^CD27^−^ cells measured by flow cytometry. (D) IL‐10 secretion in the supernatant of the indicated cord blood cell populations measured by ELISA. (E–F) Frequency of IL‐10‐producing CD19^+^CD24^hi^CD27^+^ (“B10”) cell (E) and IL‐10‐producing CD19^+^ cell (F) populations in the cord blood of infants later developing AD and in healthy infants. (G–M) Flow cytometric characterization of CD19^+^CD24^hi^CD27^+^IL‐10^+^ B10 cells in the cord blood of infants later developing AD. CBMC of infants later developing AD and of healthy controls were stimulated with CpG and CD40L for 36 h and stained for the indicated functional markers of B10 cells. C: *N* = 31; D: *N* = 3; E‐M: HC *n* = 15, AD *n* = 16. *p* values were calculated using Wilcoxon test (C), Student's *t*‐test (D, F–M), and Mann–Whitney test (E). Diagnostic certainty of AD based on 2‐year follow‐up. Abbreviation: AD: Atopic dermatitis, HC: Healthy controls. ND: Not detected.

The CD24^hi^CD27^+^ subset of CD19^+^ B cells is a key source of IL‐10, an immunoregulatory cytokine, and is commonly referred to as B10 cells [35, 44]. To investigate whether the CD24^hi^CD27^+^ subset of CD19^+^ B cells which we detected in cord blood overlap with B10 cells, we performed functional analyses on this cell type. Although IL‐10‐producing B cells are scarce in both peripheral (PBMC) and cord blood, pro‐B10 cells in peripheral blood have the potential to mature into IL‐10‐producing cells following stimulation with CpG and CD40L for 2 days [38]. Upon 36 h of stimulation of CBMC with CpG and CD40L we could detect a significant amount of IL‐10‐producing B cells (Figure 4A). Following this stimulation, we generally observed high levels of IL‐10 secretion among CD19^+^CD24^hi^CD27^+^ cord blood cells using both flow cytometric analysis and ELISA (Figure 4B–D). Although the scarcity of these cells did not allow functional analysis on an individual level, we could prove the capacity of CD19^+^CD24^hi^CD27^+^ cells to act as regulatory cells and a source of IL‐10. Upon comparing the IL‐10‐producing capacity of CD19^+^CD24^hi^CD27^+^ cells in infants later developing AD and in healthy infants, we could observe a tendency toward but no significant reduction in the former group (Figure 4E). Similarly, when IL‐10 production in all CD19^+^ B cells was compared, a tendency toward but no significant drop was found in the cord blood of infants later developing AD (Figure 4F). Thus, we could detect reduced amounts of Breg cells but no major impairment in their capacity to produce IL‐10.

### 3.7 Preserved Systemic IL‐10 Levels Despite Reduced Breg Cells

To assess whether reduced Breg‐cell numbers in the cord blood of infants later developing AD lead to altered systemic IL‐10 secretion, we quantified total IL‐10 concentrations in cord blood using ELISA (AD: *n* = 33, HC: *n* = 37) and LegendPlex assays (AD: *n* = 33, HC: *n* = 55). Total IL‐10 levels did not differ between AD patients and healthy controls (Figure S6). This finding indicates that IL‐10 production by other cells compensates for the decrease in Breg cells.

### 3.8 Cord Blood Derived B10 Cells in Infants Later Developing AD Express Regulatory Markers

As demonstrated above, we found that infants who later developed AD display reduced percentages of Breg cells in their cord blood. To assess whether the phenotype and possibly function of these cells are also altered upon environmental challenges such as activation during infections, we studied the expression of various functional markers on cord blood B10 cells using flow cytometry following 36 h of stimulation with CpG and CD40L, simulating activation by infection.

B10 cells in the cord blood of AD infants did not differ from controls with regard to the expression of CD1d, FasL, Granzyme B, PD‐1 and PD‐L1, indicating an absence of an intrinsic defect of B10‐cell function (Figure 4 G‐K). However, a higher percentage of cord blood B10 cells of AD infants expressed TGF‐β (*p* = 0.0448) and TIM‐1 (*p* = 0.0097) compared to healthy controls (Figure 4 L‐M), indicating that despite their reduced percentages in cord blood, the remaining B10 cells do not exhibit major functional impairment; but in contrast, these seem to be ready to exert suppressive functions.

### 3.9 Distinct Patterns of Breg‐Cell Development in Early‐ and Late‐Onset AD: A 3‐Year Follow‐Up

For six children who later developed AD, cord blood samples, clinical data, and 36‐month peripheral blood samples were available. Despite the low number of children, we decided to analyze the samples in more detail. Based on the timing of AD onset (before or after 6 months of age), three children were classified as having early‐onset AD and three as having late‐onset AD (Table S3). Physician's Global Assessment (PGA) scores were similar between the early‐ and late‐onset AD groups. Three children had early‐onset and three late‐onset AD. All experienced recurrent flare‐ups; five had a family history of AD. Early‐onset AD was associated with shorter breastfeeding (< 6 months). Although neither reference values nor normal frequencies are established for Breg cells in cord blood or peripheral blood, we plotted the frequencies of Breg cells first over time: first in the cord blood and then in the 3‐year peripheral blood samples.

**FIGURE 5:**
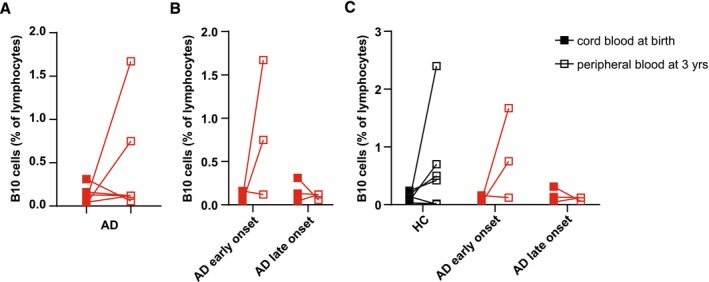
Distinct pattern of Breg‐cell frequencies in infants with early compared to late onset atopic dermatitis: A 3‐year follow‐up. (A) Frequency of CD19^+^CD24^hi^CD27^+^ Breg cells in the cord blood of infants and in the peripheral blood of children at 3 years of age who developed AD in the first 3 years of life (B) Frequency of CD19^+^CD24^hi^CD27^+^ Breg cells in the cord blood of infants and in the peripheral blood of children at 3 years of age who developed AD in the first 3 years of life, grouped based on age at onset of AD (early onset: Within the first 6 months of life, late onset: after 6 months) (C) Frequency of CD19^+^CD24^hi^CD27^+^ Breg cells in the cord blood of infants and in the peripheral blood of children at 3 years of age who were either healthy controls or developed AD in the first 3 years of life, grouped based on age at onset of AD. Abbreviation: HC *n* = 6, AD *n* = 6 (early onset *n* = 3, late onset *n* = 3). AD: Atopic dermatitis, HC: Healthy controls.

Breg‐cell frequencies showed heterogeneous patterns (Figure 5A). Grouped by AD onset, children with early‐onset AD showed increasing Breg levels over time, suggesting normalization, while levels declined in late‐onset cases (Figure 5B). In matched healthy controls, Breg frequencies increased in all but one child (Figure 5C).

Thus, our findings from the MAPS cohort indicate that reduced B10‐cell frequencies in the cord blood may serve as a biomarker for the development of AD and that monitoring B10‐cell frequencies over time may allow further stratification of AD children. However, these findings need to be confirmed in larger cohorts.

## 4 Discussion

The findings of this study underscore the interplay between genetic and environmental factors in AD pathogenesis. We identified altered immune profiles in the cord blood of infants who later developed AD, namely reduced CD4+ T cells, increased B cells, and for the first time, diminished Breg‐cell frequencies. We show that maternal allergen‐specific immunotherapy (AIT) may positively influence Breg‐cell development. To place these immune alterations in clinical context, it was essential to evaluate them alongside established epidemiological risk factors for AD. Demonstrating that our cohort reproduced well‐known perinatal and familial risk associations increases confidence that the observed immune signatures, particularly the reduction in regulatory B cells, reflect biologically relevant early‐life markers rather than cohort‐specific variation. This integrated interpretation is especially important in a relatively small cohort such as ours, where epidemiological validation supports the robustness of the immunological findings.

Genetic predisposition is a major driver of skin barrier dysfunction and immune dysregulation in AD [7, 12, 13]. Infants born to mothers with AD were found to have a threefold higher risk of developing AD within the first 6 months, whereas there was no association with paternal AD, supporting prior findings that maternal AD is a stronger predictor [61, 62]. This may relate to transplacental transfer of maternal antibodies, antigens, cytokines, and immunological factors in breast milk, contributing to a shared immune environment [63, 64, 65]. Notably, having an older sibling with AD showed the strongest association, with a nearly fivefold increased risk of early AD, consistent with prior data [14, 66]. Notably, the association between sibling AD status and disease onset appeared stronger than the association between maternal AD and infant risk [64]. This likely reflects shared genetic backgrounds and fewer diluted risk alleles [66, 67].

Environmental factors play a decisive role in AD pathogenesis [68, 69]. Our findings suggest a protective effect of cold remedy intake. Since cold remedies were not further specified in the questionnaire, no conclusion can be drawn on the effect of any specific active substance. It is likely that infants who took cold remedies had experienced a common cold. A birth cohort study of 329 children found that a respiratory infection in the first 18 months halved the risk of AD [70]. Similarly, exposure to respiratory infection during pregnancy or infancy may be protective, possibly by promoting a favorable Th1 cytokine balance that suppresses, for example, immunoglobulin E (IgE) synthesis [71, 72]. Timely microbial exposure may thus modulate the immune system to reduce the risk of AD. These findings are in line with what was addressed in the so‐called “hygiene hypothesis”: Reduced microbial exposure and less infections are linked to increased allergic disease prevalence. The inverse correlation between “hygiene” and AD risk has been widely studied and was confirmed in several analyses [73, 74].

Some known risk factors, like season of birth, were not found to be significant with regard to the occurrence of AD in our cohort. Previous studies, including one in over 16 million children, showed only a slight increase in AD risk for births in fall (OR 1.16) [75, 76]. Our sample size may not capture such small effects. The protective role of breastfeeding remains controversial. Some studies suggest it reduces the risk, particularly of eczema chronification, leading to recommendations for exclusive breastfeeding for at least 4 months [76, 77]. However, large studies, including one with over 51,000 children, found no significant association between breastfeeding and lower AD risk [78, 79]. In our study breastfeeding also had no protective effect, though it may be relevant in specific subgroups, such as in children without a positive family history of atopic diseases [80]. It is important to note that there may be a bias in participating in MAPS, as families with a history of atopic diseases may be more attracted by this offer than families without, leading to an overrepresentation in our study.

Contrary to earlier studies, we found no association between place of residence and AD risk [81, 82, 83]. However, our “rural” areas were within the Munich metro region, possibly limiting the contrast between urban and rural environments.

Infants who later developed AD showed lower CD4+ T cells, higher B cells, and lower Breg‐cell frequencies in cord blood. CD4+ T cells play a central role in AD, and data from 98 children in the LISA cohort linked reduced IFN‐γ‐producing CD4+ T cells in cord blood to a fivefold increased AD risk [84]. Consistent with our findings, CD8+ T cells showed no association with AD risk [84]. Currently, there is no data on the association of Breg cells in cord blood and AD development. Yoshihara et al. found comparable CD19^+^CD24^hi^CD38^hi^ Breg progenitor cell frequencies in adult patients with mild or severe AD and healthy controls, but IL‐10‐producing Breg (B10) cells were reduced in AD patients, with levels correlating with disease severity [49]. Importantly, despite the decrease in B10 cells, Yoshihara et al. reported no reduction in systemic IL‐10 levels [49]. This is consistent with our data showing unchanged serum IL‐10 concentrations in AD patients. Other IL‐10‐producing immune cell subsets, such as regulatory T cells, monocytes/macrophages, and dendritic cells, likely compensate for any reduction in B10‐derived IL‐10. Similarly, Gu et al. observed comparable CD19^+^CD24^hi^CD38^hi^ B cells frequencies in adults with and without AD, but again, B10 cells were reduced in AD patients [38]. Unlike Yoshihara et al., Gu et al. found no correlation between B10‐cell frequency and disease severity, which aligns with our findings. Preliminary observations in a few patients suggested that reduced B10‐cell frequencies may persist in late‐onset AD, but this needs to be confirmed in larger studies.

We found that maternal AIT is associated with increased B10‐cell frequencies in cord blood. Previous studies showed that AIT during pregnancy may modulate the fetal immune system, similar to early in utero exposures such as contact with farm animals, potentially reducing allergic sensitization [85, 86, 87]. Allergen‐specific IgG antibodies induced by AIT can cross the placenta starting from week 16–20, and their presence in cord blood has been linked to reduced atopy in offspring [88, 89]. One study of 227 children reported an 8.1% lower risk of allergic disease in those born to mothers who received AIT before or during pregnancy, though this was not statistically significant after adjusting for confounders [85, 90]. Other retrospective studies have also failed to show a clear benefit, finding similar rates of allergic rhinitis or asthma in children of AIT‐treated mothers compared to the general atopic population [91, 92, 93]. Thus, current evidence does not confirm a definitive effect of maternal AIT on AD development.

The number of B cells in cord blood is increased and their phenotypes are distinct compared to adult blood [94, 95]. Compared to other cell subsets, the role of B cells during pregnancy and early life has been less studied [96]. Notably, IL‐10‐producing Breg cells (CD24^hi^CD38^hi^) are more frequent in the cord blood of healthy neonates, likely due to the immaturity of the immune system [96]. Neonatal CD24^hi^CD38^hi^ Breg cells share phenotypic and functional similarities with adult Breg cells, suppressing IFN‐γ and IL‐4 production by T cells [40, 97, 98]. To date, factors influencing B10‐cell counts in cord blood are largely unknown, but some conditions, such as preterm birth, seem to be associated with their reduced numbers and impaired functions [99]. Further studies are needed to elucidate the development and fate of B10 cells and what factors determine their expansion and activation in general.

Breg cells have been less studied in allergic than in autoimmune diseases [49]. The transfer of splenic B10 progenitor cells has been shown to reduce skin inflammation in murine models of contact hypersensitivity [44]. In a hapten‐induced mouse model of AD, B10‐cell numbers were reduced and their ability to suppress IgE production was compromised [100]. Lee et al. have recently described an IL‐10–mediated mechanism in Bregs that mitigates AD by inhibiting eosinophil activation and tissue infiltration [101]. These findings support a key role for B10 cells in regulating allergic responses and suggest that early B10‐cell dysfunction may contribute to AD development.

Phenotypic analysis of cord blood B10 cells after in vitro activation showed no major impairment in infants who later developed AD. On the contrary, we observed a higher proportion with markers suggestive of regulatory function, namely TGF‐β and TIM‐1. This may represent a compensatory mechanism in response to their overall reduced numbers. While TIM‐1 promotes Breg‐cell expansion in mice and is essential for IL‐10 expression and the regulation of tissue inflammation, its expression is neither sufficient for IL‐10 secretion nor for suppression of T‐cell‐derived proinflammatory cytokines [102, 103, 104]. TGF‐β regulates immune responses by modulating T‐cell differentiation, proliferation, and function while also inhibiting B‐cell proliferation [105, 106]. In an EAE mouse model of multiple sclerosis, B cell‐specific TGF‐β1 deletion led to earlier disease onset, increased disease severity, and enhanced production of inflammatory cytokines, highlighting the role of TGF‐β + Bregs in immune tolerance [107]. Furthermore, TGF‐β + Bregs have also been implicated in the regulation of allergic diseases [108]. In addition, CpG‐stimulated human B cells suppress CD4+ T‐cell proliferation via TGF‐β, while promoting the Treg differentiation independently of IL‐10 [109].

Beyond cytokine production, Bregs exert their suppressive functions through CD80/86 interactions with T cells [40, 97, 110, 111]. However, due to the limited availability of infant‐derived Breg cells, we could not conduct functional assays to assess their immunomodulatory effects. Further studies are required to clarify Breg function in this context.

This study has a few limitations that should be considered. First, the number of available infant‐derived Breg cells restricted our ability to perform functional assays. Second, the longitudinal subset at 3 years of age comprised only a small number of samples, which limits the interpretability of temporal immune changes and prevented the development of a reliable predictive model. Larger cohorts with repeated sampling will be essential to integrate epidemiological and immunological markers into more comprehensive predictive frameworks.

Personalized medicine today focuses on treatment, but there is a growing need for personalized prevention based on individualized risk prediction. Our findings on reduced CD4+ T‐cell, increased B‐cell and especially diminished B10‐cell frequencies in cord blood of infants later developing AD could help identify infants who may benefit from early preventive measures, such as the timely introduction of complementary feeding, regular moisturizer use or even early introduction of targeted therapies to reduce the risk for the development of atopic diseases [112, 113, 114]. Integrating cord blood B10‐cell analysis into future, model‐based evaluation panels may therefore offer valuable insights for early risk stratification once larger datasets become available.

## 5 Conclusion

The findings of our study underscore the intricate interplay between genetic predisposition, immune system development, and environmental factors in the pathogenesis of AD in infants. Crucially, we could identify a distinct immune signature in the cord blood of infants who later develop AD, including a notable reduction in regulatory B‐cell (Breg‐cell) frequencies. Moreover, our results point to the potential of maternal allergen‐specific immunotherapy in modulating neonatal immune profiles and possibly reducing the risk of AD. In this context, the measurement of B10 cells in cord blood may offer valuable insights for early risk stratification and should be considered for inclusion in future panels aimed at personalized prevention strategies.

## Funding

Helmholtz Zentrum München, German Research Center for Environmental Health GmbH, Clinical Unit Allergology (EKA AZ2015393, 2016‐2020); Deutsche Forschungsgemeinschaft (DFG), GRK2668 (435874434); DFG BI696/14‐1 527318848; DDG‐ADF Clinician Scientist Scholarship; CRC1371.

## Conflicts of Interest

T. Biedermann gave advice to or got a honorarium for talks or research grant from the following companies: AbbVie, Alk‐Abelló, Almirall, Celgene‐BMS, Galderma, Leo Pharma, Lilly Deutschland GmbH, Mylan, Novartis, Phadia‐Thermo Fisher, Sanofi, Regeneron, Viatris. A. Zink has been an advisor and/or received speaker's honoraria and/or received grants and/or participated in clinical trials from/of the following companies: AbbVie, ALK Abello, Almirall, Amgen, Beiersdorf Dermo Medical, Bencard Allergie, BMS, Celgene, Eli Lilly, GSK, Incyte, Janssen Cilag, Leo Pharma, Miltenyi Biotec, MSD, Novartis, Pfizer, Sanofi‐Aventis, Takeda Pharma, Thermo Fisher Scientific Phadia, UCB. S. Preis received speaker's honoraria from Janssen, Novartis, Abbvie. M. Schielein is currently employed by Novartis Pharma GmbH. B. Evers has served as an advisor, received speaker honoraria, travel grants, and/or served as an investigator in clinical trials sponsored by AbbVie, Almirall, Amgen, Bristol Myers Squibb, Johnson & Johnson, LEO Pharma, Novartis, Sanofi, and UCB. The other Co‐Authors have no conflicts of interest to declare.

## Supporting information


**Data S1:** Supporting Information.

## Data Availability

The data that support the findings of this study are available from the corresponding author upon reasonable request.
