# Supplementary material for: Early Epidemiologic and Immune Predictors of Atopic Dermatitis: Reduced Cord Blood Regulatory B10 Cells in the Munich Atopy Prediction Study (MAPS)
Source: Allergy. 2026 Mar 19;81(7):2463–77. doi: 10.1111/all.70306 (PMC13342796; doi:10.1111/all.70306)
Supplement: Supplementary file 1 — Data S1: Supporting Information. [file ALL-81-2463-s001.docx]

SUPPLEMENTARY MATERIAL

Supplementary Methods:

**Subjects**

Participants were recruited at three major hospitals in Munich. Mothers 18 years or older who lived in the greater Munich area and who were able to fill out a German questionnaire were eligible for participation. There were no exclusion criteria. At prenatal recruitment, the study team obtained informed consent, administered a brief interview, and provided a take-home self-administered questionnaire about pregnancy history. At the first study visit, approximately two months after birth, at the 6 months visit and at the 2-year visit, another brief interview about the child’s (skin) health was performed and another questionnaire about the first months of life was provided. Written informed consent was obtained from the parents of each patient and all healthy controls. The inclusion criterion for the laboratory analysis was the attendance to all follow-up visits until the two-year visit.

**Measurement of Clinical Data**

Data were collected from multiple sources. At study inclusion, a baseline questionnaire recorded maternal sociodemographic data, mental health before birth, pregnancy course, smoking in the year before pregnancy, family history of AD, housing conditions, and area of residence. Maternal mental health was assessed using the German version of the Hospital Anxiety and Depression Scale (HADS) [1].

At the first visit after two months, a second questionnaire focused on anthropometric measurements, skin conditions, general health of the infant, medication, vaccination, nutrition, environmental factors, and skincare habits. In all following visits, a third questionnaire covered family environment, mental behavior of caregiving parent(s), travels, and the child’s health, including recent diseases and vaccinations [2]. Missing questionnaires were completed by phone.
Clinical examinations at two months, six months, and two years included interviews about recent skin diseases, allergies, therapies, and diagnostics, as well as skin inspections assessing skin type, nevi, hair, nails, and benign skin changes (e.g., freckles, accessory mammillae) [2]. The skin was evaluated for AD using Hanifin and Rajka criteria (excluding IgE levels) [3]. If AD was diagnosed, severity was assessed using EASI and SCORAD. Infants missing the two-year visit were excluded due to unreliable AD status.

At the three-year visit, peripheral blood was collected, and an ImmunoCAP ISAC test (Thermo Fisher Scientific, Waltham, Massachusetts, USA) was performed. The same questionnaire as in previous visits was administered.

**PBMC Preparation and Flow Cytometry**

Immediately after birth, 80 ml of blood was collected from the umbilical cord veins of newborns into a Vita 34 collection bag (Vita 34 AG, Leipzig, Germany). Peripheral blood mononuclear cells (PBMCs) were isolated by Ficoll gradient centrifugation (Sigma-Aldrich, St. Louis, MO), washed, and resuspended in phosphate-buffered saline (PBS; Biowest, Nuaillé, France). Cells were stained with monoclonal antibodies (Biolegend, San Diego, USA; BD Biosciences, New Jersey, USA; eBioscience, Frankfurt am Main, Germany) for immunophenotyping:

- **Dendritic cells (DCs):** CD11c-FITC, CD123-PE, HLA-DR-APC, CD1c-PB, CD14-PerCp-Cy5.5, Lin1-BV510.
- **Mast cell progenitors:** CD34-PerCP, CD133-PE, CD13-APC, CD117-FITC, Lin1-BV510.
- **B cells:** CD19.
- **B10 cells:** CD19 PerCp-Cy5.5, CD23 PeC7, CD27-PB, CD24-APC, CD148-PE, CD48-FITC.
- **Myeloid-derived suppressor cells (MDSCs):** CD14-PerCP-Cy5.5, CD11b-FITC, CD33-PE, HLA-DR-APC, Lin1-BV510.
- **Neutrophils:** CD66b, CD33, CD123 (HLA-DR-negative).
- **T cells:** CD4-PB, CD8-PerCP, CD3-FITC, αβ-TCR-APC, γδ-TCR-PE-Cy7.

Isotype controls were included to verify antibody specificity. Samples were analyzed using FACSCanto II (BD Biosciences, New Jersey, USA). Remaining PBMCs were cryopreserved in liquid nitrogen for subsequent analyses.

**B10 Cell Culture**

Frozen PBMCs were thawed and cultured in complete RPMI 1640 medium (Sigma-Aldrich) with 10% fetal calf serum, 110 U/ml penicillin, 110 mg/ml streptomycin, and 0.3231 mg/ml L-glutamine (Gibco, Auckland, New Zealand). Cells were stimulated with CpG/ODN 2006 (10 µg/mL; InvivoGen, San Diego, CA, USA) and CD40L (1 µg/mL, R&D Systems, Minneapolis, MN, USA) for 48 h. PMA (50 ng/mL) and ionomycin (1 µg/mL) were added for the final 5 h along with Golgi Stop™ (0.2 μL; BD Biosciences). After culture, extracellular staining was performed (CD19-BV510, CD24-PerCP, CD27-PE), followed by fixation, permeabilization, and intracellular IL-10-APC staining. Isotype controls were used to confirm specificity.

**IL-10 Secretion Assay**

PBMCs were stimulated for 36 hours as described, sorted for CD19+CD27^hi^CD24+ cells using BD FACSAria™ Fusion (BD Biosciences), and re-cultured for an additional 24 h. PMA (60 ng/mL) and ionomycin (82 ng/mL) were added for the last 5 hours. Secreted IL-10 was quantified using ELISA kits (Biolegend) per manufacturer instructions.

**B10 Cell Characterization**

Cord blood PBMCs were stimulated with CpG and CD40L as above. For surface markers, cells were stained with LD-APC-EF780, CD19-BV510, CD24-PerCP, CD27-PB, PDL1-BV711, TIM1-BV605, CD1d-BV395, FAS-L-BV650, PD1-PECy7. Intracellular staining was done using TGF-β-FITC, Granzyme B-PE, and IL-10-APC. B10 cells were defined as CD19+ IL-10+ and further gated based on surface marker expression.

**Statistical Analysis**

Study data were collected and managed using REDCap electronic data capture tools hosted at the Department of Dermatology and Allergy, TUM [4]. To identify and eliminate potential errors in manual data digitalization, 10% of questionnaires were entered twice (error rate 0.31%) and any discrepancies between the resulting datasets were corrected. Infant birth months were grouped as spring-summer (March-August) and autumn-winter (September-February). Breastfeeding duration was categorized as <6 months or ≥6 months per WHO guidelines. Education level was classified using ISCED 2011. HADS subscale scores ≥8 were considered positive for anxiety or depression.

Characteristics of the infants were statistically compared by AD status using the Pearson Chi 2 test. Fisher’s exact test was used when the expected frequency for any cell was less than 5. To identify predictive factors of infant AD at 6 months of life, univariate logistic regression using the firth method was conducted for all variables separately and results are presented as unadjusted Odds Ratios (OR) for the original dataset which excluded infants with missing data.

The firth procedure is the current method of choice to reduce bias in logistic regression in small samples and in tables with zero cell counts using a penalized maximum likelihood estimation [5, 6]. Data was identified to be missing at random and chained equations were used to impute missing categorical data [7]. Overall, a total of ten imputations was generated to account for missing data. Univariate firth logistic regression was performed for the multiply imputed dataset (MI). Point estimates and confidence intervals (CI) were then calculated by the combination of penalized likelihood profiles (CLIP) described by Heinze, Ploner and Beyea [8]. This method is useful for computing CI for parameters after multiple imputation of small data sets. The imputed dataset was used for the multivariate analyses. Multiple firth logistic regression was conducted to evaluate the combined effect of variables with a p‑value < 0.100 in the univariate analysis. The final multivariate model was built by adding variables considered a priori as potential confounders relating to infant AD. Analysis of multicollinearity among the predictors was performed by calculating the phi coefficient (Φ). Pairs of variables that showed a strong correlation (|Φ|≥0.30) were not included into the multivariate model simultaneously. Results from multiple logistic regression are presented as adjusted ORs (aOR) with CI at α=0.05.

For all conducted analyses, testing was two-sided, the level of significance was α=0.05 and 95% CIs are provided. Descriptive statistics and data preparation was performed using IBM SPSS Statistics Version 26 (IBM Corporation, Armonk, NY, USA). R software (version 4.0.4, www.r-project.org) was used for logistic regression models.

Experimental data were analyzed using GraphPad Prism 7. Normality was assessed via Kolmogorov-Smirnov test. Non-parametric data were analyzed using the Mann-Whitney U-test; parametric data via t-test. P-values <0.05 were considered statistically significant.

Supplementary Figure 1: Flow chart of included infants


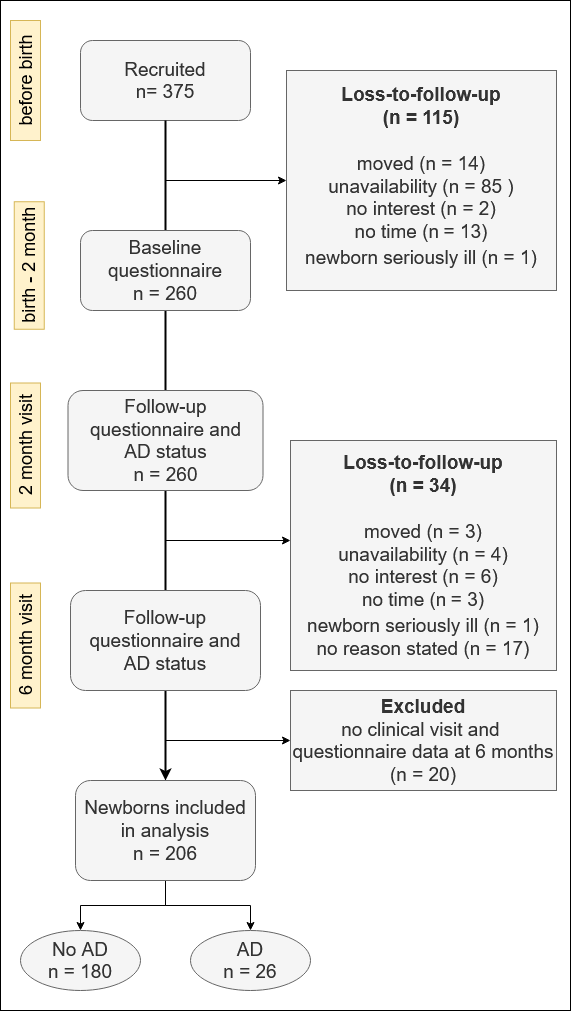


AD: atopic dermatitis

Supplementary Figure 2: Risk and protective factors for developing AD in the first 6 months of life

AD: atopic dermatitis, aOR: adjusted odds ratio

Supplementary Figure 3: The flow cytometric gatings used throughout our study is shown. FMO: fluorescence minus one, cDC: myeloid conventional dendritic cells pDCs: plasmacytoid dendritic cells, M-MDSCs: monocytic myeloid-derived suppressor cells, E-MDSCs: early stage myeloid-derived suppressor cells, PMN-MDSC: Polymorphonuclear myeloid-derived suppressor cells myeloid-derived suppressor cells


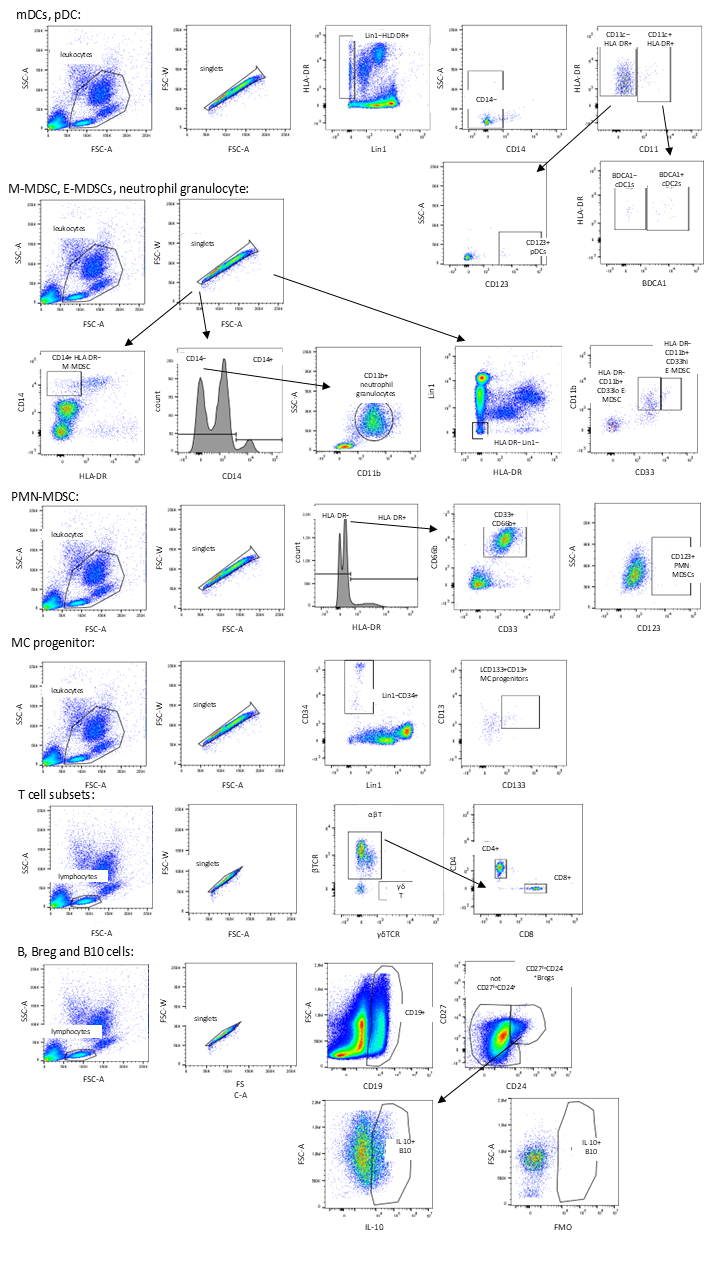


Supplementary Figure 4: CD4+ T cell subsets are unchanged in newborns who later develop AD.

Flow cytometric analysis of CD45+CD4+ T cell subsets in the cord blood of children later developing AD (n=12) and of healthy controls (n=12)

P values were calculated using Student’s t-test, diagnostic certainty of AD based on 2-year follow-up

AD: atopic dermatitis, HC: healthy controls. n.s.: not significant

*
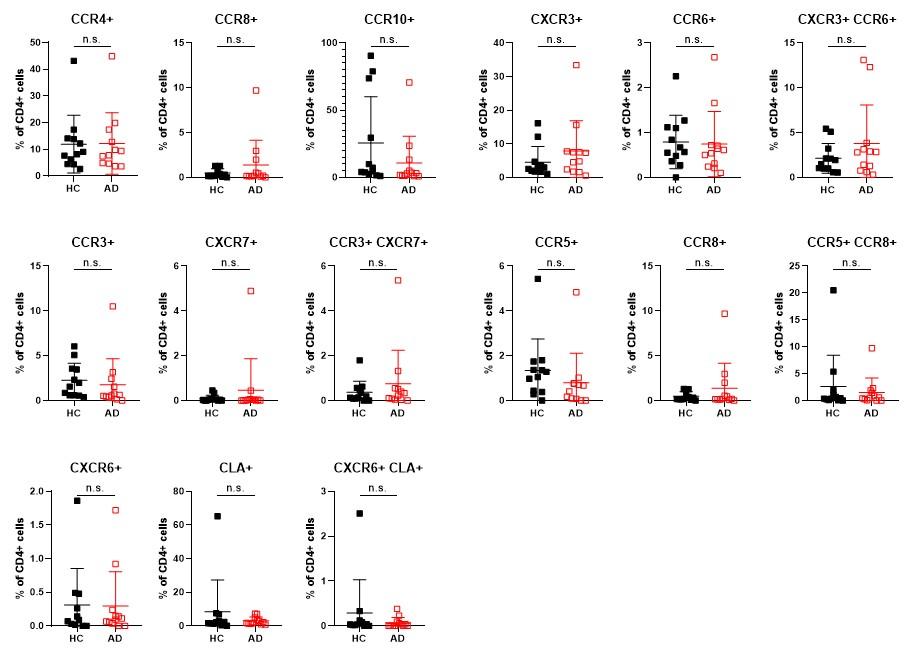
*

Supplementary Figure 5: Cord blood IgE levels are unchanged in newborns who later develop AD.

IgE Levels were measured by ELISA (AD n = 13, HC n = 28).

P values were calculated using Student’s t-test, diagnostic certainty of AD based on 2-year follow-up

AD: atopic dermatitis , HC: healthy controls. n.s.: not significant

*
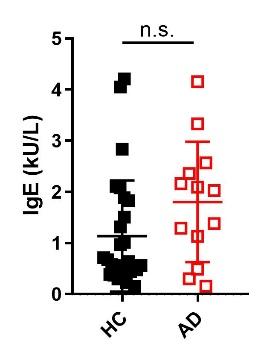
*

Supplementary Figure 6: Total IL-10 levels are maintained despite reduced frequencies of IL-10-producing B10 cells in AD. Cord blood serum IL-10 concentrations were quantified by ELISA (AD n = 33, HC n = 37) and LegendPlex cytokine analysis (AD: n = 33, HC: n = 55).

P values were calculated using Student’s t-test, diagnostic certainty of AD based on 2-year follow-up

AD: atopic dermatitis, HC: healthy controls. n.s.: not significant

*
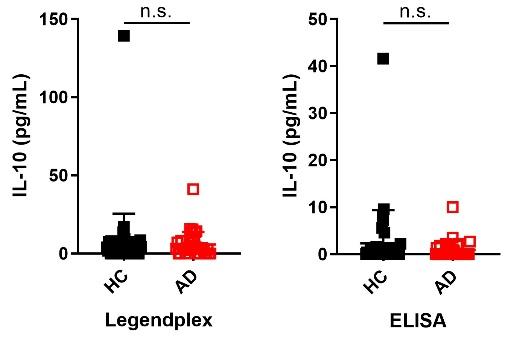
*

Supplementary Table 1: Characteristics of the infants stratified by the development of AD within the first six months of life

|  | Total  (n = 206)  n (%) | No AD  (n = 180)  n (%) | AD  (n = 26)  n (%) | p-value |
| --- | --- | --- | --- | --- |
| Sex |  |  |  |  |
| *female* | 93 (45.1) | 82 (45.6) | 11 (42.3) | 0.756 |
| *male* | 113 (54.9) | 98 (54.4) | 15 (57.7) |  |
| Birth season |  |  |  |  |
| *spring-summer* | 82 (39.8) | 75 (41.7) | 7 (26.9) | 0.151 |
| *autumn-winter* | 124 (60.2) | 105 (58.3) | 19 (73.1) |  |
| Delivery via C-section |  |  |  |  |
| *no* | 112 (54.9) | 94 (52.8) | 18 (69.2) | 0.116 |
| *yes* | 92 (45.1) | 84 (47.2) | 8 (30.8) |  |
| Older siblings |  |  |  |  |
| *no* | 139 (67.5) | 124 (68.9) | 15 (57.7) | 0.255 |
| *yes* | 67 (32.5) | 56 (31.1) | 11 (42.3) |  |
| Total duration of breastfeeding |  |  |  |  |
| *< 6 months* | 63 (32.8) | 57 (34.1) | 6 (24.0) | 0.314 |
| *≥ 6 months* | 129 (67.2) | 110 (65.9) | 19 (76.0) |  |
| Antibiotic intake during first 6 months |  |  |  |  |
| *no* | 179 (94,2) | 155 (94.5) | 24 (91.7) | 0.649 |
| *yes* | 11 (5.8) | 9 (5.5) | 2 (8.3) |  |
|  |  |  |  |  |
| Analgesics intake during first 6 months |  |  |  |  |
| *no* | 137 (62.1) | 120 (63.2) | 17 (65.4) | 0.411 |
| *yes* | 53 (27.9) | 44 (26.8) | 9 (34.6) |  |
| Cold remedy intake during first 6 months |  |  |  |  |
| *no* | 168 (88.4) | 142 (86.6) | 26 (100.0) | **0.048** |
| *yes* | 22 (11.6) | 22 (13.4) | 0 (0.0) |  |
| Vaccinations during first 6 months |  |  |  |  |
| *no* | 7 (3.6) | 7 (4.1) | 0 (0.0) | 0.597 |
| *yes* | 189 (96.4) | 163 (95.9) | 26 (100.0) |  |
| Maternal Education |  |  |  |  |
| *< 12 years* | 39 (19.1) | 32 (18.0) | 7 (26.9) | 0.290 |
| *≥ 12 years* | 165 (80.9) | 146 (82.0) | 19 (73.1) |  |
| Maternal employment before maternity leave |  |  |  |  |
| *no* | 22 (10.7) | 20 (11.1) | 2 (7.7) | 1.000 |
| *yes* | 184 (89.3) | 160 (88.9) | 24 (92.3) |  |
| Maternal AD |  |  |  |  |
| *no* | 165 (80.1) | 149 (82.8) | 16 (61.5) | **0.011** |
| *yes* | 41 (19.9) | 31 (17.2) | 10 (38.5) |  |
| Paternal AD |  |  |  |  |
| *no* | 192 (93.2) | 167 (92.8) | 25 (96.2) | 1.000 |
| *yes* | 14 (6.8) | 13 (7.2) | 1 (3.8) |  |
| Sibling with AD |  |  |  |  |
| *no* | 196 (95.1) | 174 (96.7) | 22 (84.6) | **0.025** |
| *yes* | 10 (4.9) | 6 (3.3) | 4 (15.4) |  |
| Maternal HADS-D |  |  |  |  |
| *< 8* | 171 (92.4) | 150 (92.0) | 21 (95.5) | 1.000 |
| *≥ 8* | 14 (7.6) | 13 (8.0) | 1 (4.5) |  |
| Maternal HADS-A |  |  |  |  |
| *< 8* | 142 (76.8) | 126 (77.3) | 16 (70.0) | 0.634 |
| *≥ 8* | 43 (23.2) | 37 (22.7) | 6 (30.0) |  |
| Maternal smoking during the year before pregnancy |  |  |  |  |
| *no* | 171 (83.0) | 146 (81.1) | 25 (96.2) | 0.089 |
| *yes* | 35 (17.0) | 34 (18.9) | 1 (3.8) |  |
| Maternal chemical hair treatment during pregnancy |  |  |  |  |
| *no* | 114 (55.3) | 97 (53.9) | 17 (65.4) | 0.270 |
| *yes* | 92 (44.7) | 83 (46.1) | 9 (34.6) |  |
| Maternal cold during pregnancy |  |  |  |  |
| *no* | 177 (56.8) | 105 (58.3) | 12 (46.2) | 0.241 |
| *yes* | 89 (43.2) | 75 (41.7) | 14 (53.8) |  |
| Maternal acute febrile disease during pregnancy |  |  |  |  |
| *no* | 195 (94.7) | 171 (95.0) | 24 (92.3) | 0.634 |
| *yes* | 11 (5.3) | 9 (5.0) | 2 (7.7) |  |
| Residence |  |  |  |  |
| *urban* | 143 (69.4) | 129 (71.7) | 14 (53.8) | 0.065 |
| *rural* | 63 (30.6) | 51 (28.3) | 12 (46.2) |  |
| Living close to traffic road |  |  |  |  |
| *no* | 117 (56.8) | 103 (57.2) | 14 (53.8) | 0.745 |
| *yes* | 89 (43.2) | 77 (42.8) | 12 (46.2) |  |
| Pet owner (cat/dog) |  |  |  |  |
| *no* | 169 (82.0) | 146 (81.1) | 23 (88.5) | 0.584 |
| *yes* | 37 (18.0) | 34 (18.9) | 3 (11.5) |  |

AD: atopic dermatitis; HADS-A: Hospital Anxiety and Depression Scale Anxiety subscale; HADS-D, Hospital Anxiety and Depression Scale Depression subscale

bold values showed significance at alpha 0.05. p-value for Pearson Chi square test or Fisher’s exact test (< 5 cases)

Supplementary Table 2: Potential predictive factors for AD in infants

|  | Univariate logistic regression_MI_  n=206 | | Univariate logistic regression_OD_ | |
| --- | --- | --- | --- | --- |
|  | OR (95% CI) | p-value | OR (95% CI) | p-value |
| Sex (male) | 1.13 (0.50 - 2.61) | 0.771 | 1.13 (0.50 - 2.61) | 0.771 |
| Birth season  (spring-summer) | 1.86 (0.79 - 4.82) | 0.159 | 1.86 (0.79 - 4.82) | 0.159 |
| C-Section  (yes) | 0.51 (0.21 - 1.19) | 0.121 | 0.51 (0.21 - 1.19) ^†^ | 0.121 |
| Older siblings  (yes) | 1.64 (0.70 - 3.72) | 0.248 | 1.64 (0.70 - 3.72) | 0.248 |
| Breastfeeding  (≥ 6 months) | 1.68 (0.69 - 4.63) | 0.259 | 1.56 (0.64 - 4.32) ^‡^ | 0.342 |
| Antibiotic intake  (no) | 1.57 (0.29 – 5.98) | 0.560 | 1.670 (0.31 - 6.36) ^├^ | 0.508 |
| Analgesics intake  (no) | 1.49 (0.61 - 3.48) | 0.370 | 1.47 (0.60 - 3.42) ^├^ | 0.388 |
| Cold remedy intake  (no) | 0.12 (0.001 - 0.89) | **0.035** | 0.12 (0.001 - 0.91) ^├^ | **0.038** |
| Vaccinations  (no) | 2.29 (0.27 - 300.55) | 0.527 | 2.43 (0.28 - 318.96) ^Φ^ | 0.495 |
| Maternal education  (≥ 12 years) | 0.57 (0.23 - 1.52) | 0.249 | 0.58 (0.24 - 1.53) ^†^ | 0.257 |
| Maternal employment before maternity leave (No) | 1.25 (0.37 - 6.51) | 0.744 | 1.25 (0.37 - 6.51) | 0.744 |
| Maternal AD (No) | 3.02 (1.24 - 7.11) | **0.015** | 3.02 (1.24 - 7.11) | **0.015** |
| Paternal AD (No) | 0.73 (0.08 - 3.20) | 0.712 | 0.73 (0.08 - 3.20) | 0.712 |
| Siblings with AD (No) | 5.37 (1.39 - 19.18) | **0.017** | 5.37 (1.39 - 19.18) | **0.017** |
| Maternal HADS-D ≥ 8 | 0.79 (0.09 - 3.37) | 0.788 | 0.79 (0.08 - 3.46) ^Δ^ | 0.772 |
| Maternal HADS-A ≥ 8 | 1.39 (0.50 - 3.51) | 0.503 | 1.33 (0.47 - 3.41) ^Δ^ | 0.575 |
| Maternal smoking during the year before pregnancy (No) | 0.25 (0.03 - 1.02) | **0.053** | 0.25 (0.03 - 1.02) | **0.053** |
| Chemical hair treatment | 0.63 (0.26 – 1.45) | 0.282 | 0.63 (0.26 – 1.45) | 0.282 |
| Cold during pregnancy | 1.62 (0.72 - 3.70) | 0.243 | 1.62 (0.72 - 3.70) | 0.243 |
| Acute febrile disease during pregnancy | 1.84 (0.34 - 7.01) | 0.435 | 1.84 (0.34 - 7.01) | 0.435 |
| Urban area (No) | 0.46 (0.20 - 1.06) | **0.069** | 0.46 (0.20 - 1.06) | **0.069** |
| Living next to traffic road | 1.15 (0.50 - 2.60) | 0.734 | 1.15 (0.50 - 2.60) | 0.734 |
| Cat in home | 1.02 (0.19 - 3.55) | 0.981 | 1.02 (0.19 - 3.55) | 0.981 |
| Dog in home | 0.41 (0.05 - 1.73) | 0.257 | 0.41 (0.05 - 1.73) | 0.257 |

AD: atopic dermatitis; OR: odds ratio; CI: confidence interval

HADS-D: Hospital Anxiety and Depression Scale Depression subscale.

HADS-A: Hospital Anxiety and Depression Scale Anxiety subscale.

bold values showed p-values < 0.100.

MI: multiple imputation; OD: original dataset. †: n=204 ‡: n=192 ├: n=190 Φ: n=196 Δ: n=185.

Supplementary Table 3: Characteristics of infants with AD at the age of 3 years

| Baby | FA positive for AD | Age of Onset AD (months) | Total-IgE | ISAC (species-specific components) | PGA | Pet | Breast feeding |
| --- | --- | --- | --- | --- | --- | --- | --- |
| Early AD Onset (<6 months) | | | | | | | |
| 1 | yes | 3 | 55.0 | - | 3 | yes | yes |
| 2 | no | 6 | 15.2 | - | 3 | no | yes |
| 3 | yes | 4 | 1238 | Gal d2 (1) Ana o3 (11), Cor a9 (0.4), Cor a14 (0.7), Jug r1 (1.4), Ara h 1 (20), Ara h 2 (58), Ara h3 (0.5), Ara h6 (24), Gly m6 (0.3), Tri a14 (0.4) | 2 | no | yes |
| Late AD onset (> 6 months) | | | | | | | |
| 4 | yes | 27 | 0 | - | 2 | yes | yes |
| 5 | yes | 9 | 19.5 | - | 3 | no | no |
| 6 | yes | 9 | 11.9 | - | 3 | no | no |

*breast feeding for less than six months

FA: family anamnesis

References:

1. Spinhoven, P., et al., *A validation study of the Hospital Anxiety and Depression Scale (HADS) in different groups of Dutch subjects.* Psychol Med, 1997. **27**(2): p. 363-70.

2. Preis, S., et al., *Munich atopy prediction study (MAPS): protocol for a prospective birth cohort addressing clinical and molecular risk factors for atopic dermatitis in early childhood.* BMJ Open, 2022. **12**(9): p. e059256.

3. Böhme, M., et al., *Hanifin's and Rajka's minor criteria for atopic dermatitis: which do 2-year-olds exhibit?* J Am Acad Dermatol, 2000. **43**(5 Pt 1): p. 785-92.

4. Harris, P.A., et al., *Research electronic data capture (REDCap)--a metadata-driven methodology and workflow process for providing translational research informatics support.* J Biomed Inform, 2009. **42**(2): p. 377-81.

5. Heinze, G. and M. Schemper, *A solution to the problem of separation in logistic regression.* Stat Med, 2002. **21**(16): p. 2409-19.

6. FIRTH, D., *Bias reduction of maximum likelihood estimates.* Biometrika, 1993. **80**(1): p. 27-38.

7. White, I.R., P. Royston, and A.M. Wood, *Multiple imputation using chained equations: Issues and guidance for practice.* Stat Med, 2011. **30**(4): p. 377-99.

8. Heinze, G., M. Ploner, and J. Beyea, *Confidence intervals after multiple imputation: combining profile likelihood information from logistic regressions.* Stat Med, 2013. **32**(29): p. 5062-76.
